# Thriving against the odds through positive deviant behavior: technology adoption and entrepreneurship among dairy farmers in Addis Ababa and Oromia in Ethiopia

**DOI:** 10.3389/fvets.2025.1690335

**Published:** 2026-01-21

**Authors:** Ndungu Nyokabi, Gizachew Gemechu, Lisette Phelan, Johanna Lindahl, Adane Mihret, Stefan Berg, James L. N. Wood, Henrietta L. Moore

**Affiliations:** 1 Institute for Global Prosperity, University College London, London, United Kingdom; 2 University of Edinburgh Business School, Edinburgh, United Kingdom; 3 Armauer Hansen Research Institute (AHRI), Addis Ababa, Ethiopia; 4 School of Sustainable Food and Farming, Harper Adams University, Edgmond, United Kingdom; 5 International Livestock Research Institute (ILRI), Nairobi, Kenya; 6 Department of Medical Biochemistry and Microbiology, Uppsala University, Uppsala, Sweden; 7 Department of Clinical Sciences, Swedish University of Agricultural Sciences, Uppsala, Sweden; 8 Bernhard Nocht Institute for Tropical Medicine, Hamburg, Germany; 9 Department of Veterinary Medicine, University of Cambridge, Cambridge, United Kingdom

**Keywords:** animal health, animal welfare, biosecurity measures, livestock housing, livestock production, livestock feeding, technology adoption

## Abstract

Positive deviant (PD) farmers can be differentiated from the wider farming community by their inherent capacity to leverage farming innovations and technologies in addressing challenges faced in engaging in agricultural production. There is currently a limited body of literature on how positive deviance and entrepreneurial behavior allow some dairy farmers to develop strategies that enable them to cope better with and creatively overcome challenges faced by their peers. This study employed a positive deviance approach to identify innovative dairy farmers in urban and peri-urban areas of the Addis Ababa and Oromia administrative regions of Ethiopia. PD farmers were identified and selected through a descriptive study design, utilizing a purposive and snowball sampling approach based on the number of technologies adopted in a previous survey study and referrals from other farmers. Data were collected through key informant interviews and participant observation on selected farms. We observed that PD dairy farmers had adopted and/or modified a variety of technologies to overcome context-specific challenges faced, such as seasonal feed shortages exacerbated by climate change, reduced land size and availability of land for grazing and waste disposal, and restrictions on farming resulting from the development of urban areas. These technologies enabled farmers to improve feed production, manure disposal, breeding practices, the quality of livestock housing, and animal welfare and enabled them to control diseases and add value to milk production. This study underscores the important role that PD dairy farmers could play as social referents, not only for their peers in urban and peri-urban areas but also for policymakers, extension workers and academics who are interested in working with dairy farmers in co-identifying and co-developing solutions to challenges currently undermining the sustainability of the dairy sector in Ethiopia and beyond.

## Introduction

Agriculture plays an important societal and economic function, contributing toward food security and rural development across countries in Africa (1). Smallholder dairy production, in particular, is an important income and livelihood source in East Africa (2–4). Adoption of technologies by smallholder dairy farming households’ provides them with opportunities to improve household income, milk production, food safety, animal health and welfare (4–8). Demographic, socioeconomic and institutional factors influence dairy farmers adoption and use of technologies, as do their social networks, membership in livestock cooperatives and farmer groups, which also provide farmers with the opportunities to access knowledge and training regarding technologies (3, 7–9). Although classical, top-down approaches to technology and innovation diffusion have long led dairy farmers to adopt ‘techno-centric fixes’ developed by external actors (10). However, studies have also shown that some entrepreneurial and innovative dairy farmers address challenges they face in pursuing their livelihood strategy by designing, deploying and/or implementing context-specific solutions that they have devised themselves, using available resources and information (5, 11). These entrepreneurial and innovative farmers can be conceptualized as “positive deviant (PD) farmers” and are often considered social referents by their peers (4, 5).

Research has shown that viable solutions exist within farming communities, particularly among PD farmers who can innovate and modify technologies to fit their contextual needs (4, 5, 10). Positive deviance is an asset-based approach to developing best-fit technologies, innovations and solutions, that are socially desirable, technologically feasible, politically acceptable and economically viable that reflects local contextual factors and builds on local knowledge systems. Research focused on positive deviance behavior at an individual farm level contributes to the identification and amplification of local solutions that exist within a community (12, 13). In a dairy farming context, positive deviance equates to more than standard animal husbandry practices; it includes practices and technologies that have been learned experientially and through experimentation, involving a trial and error approach, as well as from neighbors and relatives, extension services, and research projects (13). In East Africa, smallholder PD farm households have been reported to achieve higher productivity and income levels through intensive dairy farming practices, utilizing more farm inputs and services, and marketing their milk through formal and informal milk marketing channels (4, 5). PD farmers provide their cows with supplementary feeding, improved housing, vaccination, deworming and improved animal health and welfare (13).

This paper uses Ethiopia as a case study to explore how dairy production challenges can be addressed through solutions developed by PD farmers and adopted by the wider smallholder dairy farming community in East Africa. There are several reasons for doing so. Firstly, smallholder dairy production is important for farmers’ livelihoods, particularly women and youth (3, 14). Secondly, livestock production challenges such as low production levels, information asymmetry, low milk prices, livestock diseases, poor animal welfare and urbanization continue to undermine the development of smallholder dairy production systems (15–19). Thirdly, there is a need to improve livestock production to supply the growing demand for livestock goods such as milk and meat, driven by the fast growth of the Ethiopian economy and improved household incomes (3). Finally, although a handful of studies have explored the adoption of dairy farming technologies by smallholder farmers in Ethiopia (3, 7, 8), few studies have investigated and documented the adoption and use of technologies and innovations developed by farmers rather than external actors. The main objective of this study was, therefore, to document PD farmers’ entrepreneurial behavior supports the growth of dairy farming and the adoption of dairy practices and technologies in urban and peri-urban areas of Addis Ababa and the Oromia region of Ethiopia.

## Methodology

This study was conducted in the context of the Ethiopia Control of Bovine Tuberculosis Strategies (ETHICOBOTS) research project that focused on the control of bovine tuberculosis in smallholder dairy systems.^1^ The study builds on previous research that explored the adoption of dairy technology (8), biosecurity practices (7, 20) and food safety measures (21) by smallholder dairy farmers in urban and peri-urban areas in Ethiopia. The above-referenced research explored smallholder dairy farmers’ technology adoption and the intensity and drivers of technology adoption, through a quantitative approach. While this quantitative approach facilitated the categorization of farmers in Ethiopia as high, medium and low adopters of technologies and innovations, these previous studies did not reveal who the well-performing farmers (i.e., PD farmers) were, what dairy farming technologies they had adopted, and what factors facilitated their adoption of these innovations that led them to have more profitable farms relative to their peers (i.e., non-PD farmers) (3, 5). The present study employs a research method frequently used in positive deviance studies, namely, a qualitative ethnographic research approach, to document PD farmers’ development and adoption of technologies and innovations, and the socioeconomic, personal and contextual drivers of their innovation and use of technologies in urban and peri-urban areas of Addis Ababa and the Oromia region of Ethiopia.

### Study area

The study area encompassed the urban and peri-urban areas of Addis Ababa and the Oromia administrative regions of Ethiopia. This area is an important milk-producing region that supplies the capital city of Addis Ababa. The study locations included Bole, Kolfte, Kaliti and Ketema sub-cities of Addis Ababa and Sebeta, Holeta, Sandafa and Debre Zeit in the Oromia federal region. Travel and research plans for the lead researcher to other regions covered by the ETHICOBOTS research project were constrained by COVID-19 pandemic-related restrictions on organizing large group meetings and travel restrictions related to the civil war in northern Ethiopia.

### PD farmer selection

PD farmers were identified and selected through a purposive and snowballing sampling approach from an initial list of 159 farmers who had participated in a survey related to the in-farm adoption of dairy technologies, food safety measures and biosecurity measures. From the list, a total of 33 PD farmers were selected and included in this study. The inclusion criteria for the present study were: that the farmers had participated in this previous research related to their adoption of dairy technology, biosecurity practices and food safety measures (7, 8, 14, 20, 21); had adopted a demonstrably higher number of technologies and innovations relative to their peers, and were reported by fellow PD farmers and/or peer non-PD farmers that they regularly provided recommendations to their peers regarding technologies and innovations.

### Research design and data collection

Data relating to the technologies that PD farmers had adopted, and/or modified to fit their specific context, were collected through qualitative, open-ended, in-depth, semi-structured key-informant interviews. The key informant interviews took 30–45 min and were conducted in the local language, Amharic, in Addis Ababa and in the Afaan Oromo language in the Oromia federal region. Questions posed during the interviews focused on the adoption of dairy farming technologies. Informed verbal consent was obtained before the start of the key informant interviews from the PD farmers, with farmers briefed in the presence of a witness (a local expert) that the interviews would be recorded, their participation in this study was voluntary, and their confidentiality would be maintained. Participant observations were also conducted on 15 of the 33 dairy farms included in the study to observe and understand why and how adopted technologies and innovations had been deployed by farmers to address challenges faced in pursuing their livelihood strategy.

### Ethics clearance

This research has ethical clearance from University College London’s Research Ethics Committee (UCL-REC), approval number 19867/001, as well as from the Armauer Hansen Research Institute (AHRI) and the All-Africa Leprosy, Tuberculosis, and Rehabilitation Training Centre (ALERT) hospital. The study also had AHRI/ALERT Ethics Review Committee (AAERC) approval (Protocol number PO-46/14).

### Data analysis

The recorded key informant interviews were transcribed and translated, with the transcripts checked against the original recording to ensure that no meaning was lost during transcription and translation. Thematic content analysis was performed using NVivo®. The transcripts were thoroughly read and coded based on themes aligned with the original key informant interview schedule, while supporting quotes were identified to support the main findings. The findings of the participant observations made at the farm level were compared with the findings of the key informant interviews to ensure the consistency and accuracy of the analysis.

## Results

### Farm characteristics

PD farmers practiced intensive dairy farming characterized by highly productive exotic breeds and their crosses, improved cattle housing, improved feeding strategies, fodder cultivation, and feed conservation strategies. Moreover, they had incorporated mechanization, including milking machines, fodder choppers and feed mixing machines. Four of the PD farmers had employed an in-house animal health professional to ensure that they maintained high production levels by minimizing disease incidents.

### Technologies adopted and modified by PD farmers

**Figure 1:**
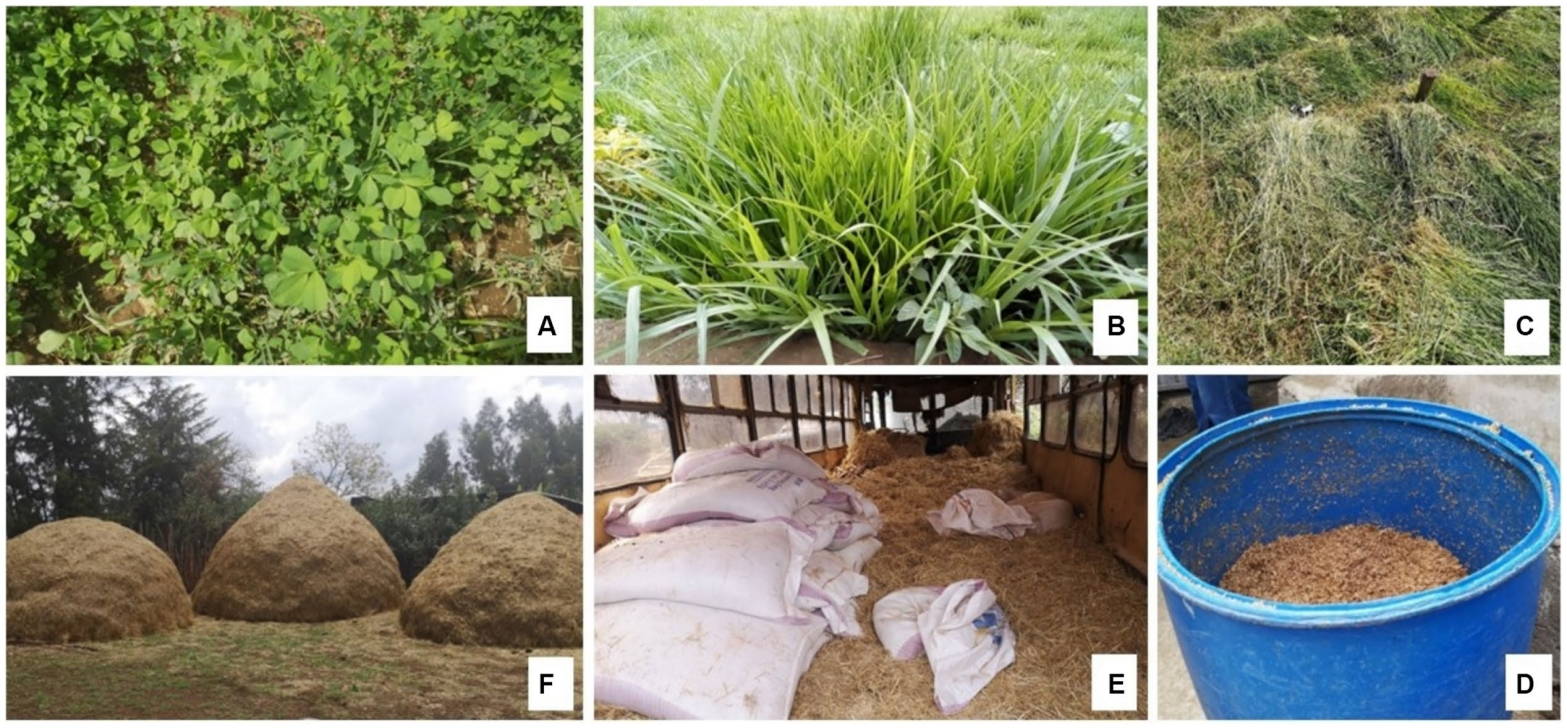
Cattle feed production and conservation practices. **(A)** Planted clover; **(B)** Planted Napier grass; **(C)** Harvested grass for livestock feeding; **(D)** Mixing cattle rations; **(E)** Purchased commercial dairy meal; **(F)** Traditional teff stover storage technique.

**Figure 2:**
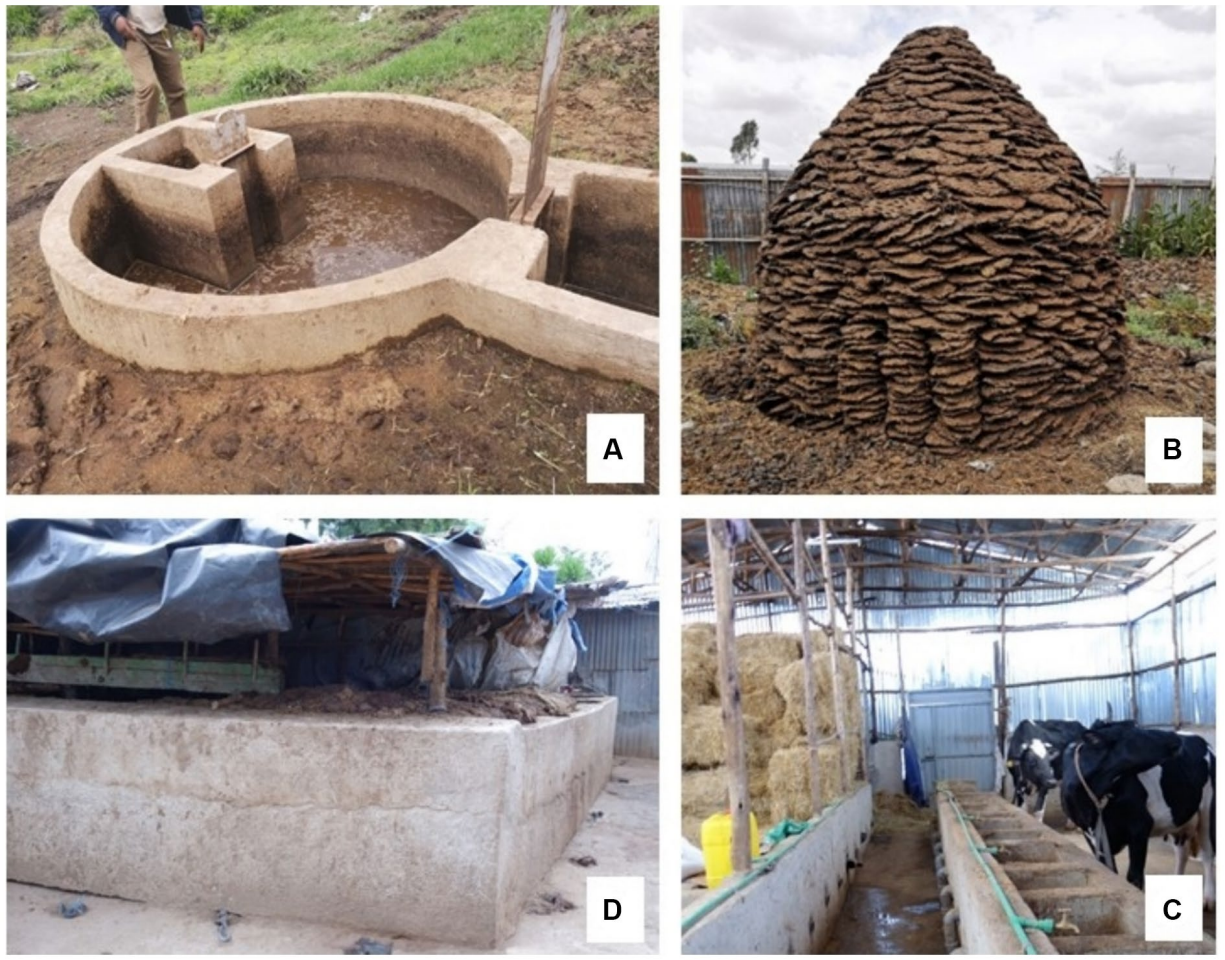
Innovative cattle waste management practices and improved housing and feeding troughs. **(A)** Biogas plant; **(B)** Cow dung patties used as fuel; **(C)** Manure storage pit; **(D)** Improved housing and feeding troughs.

**Figure 3:**
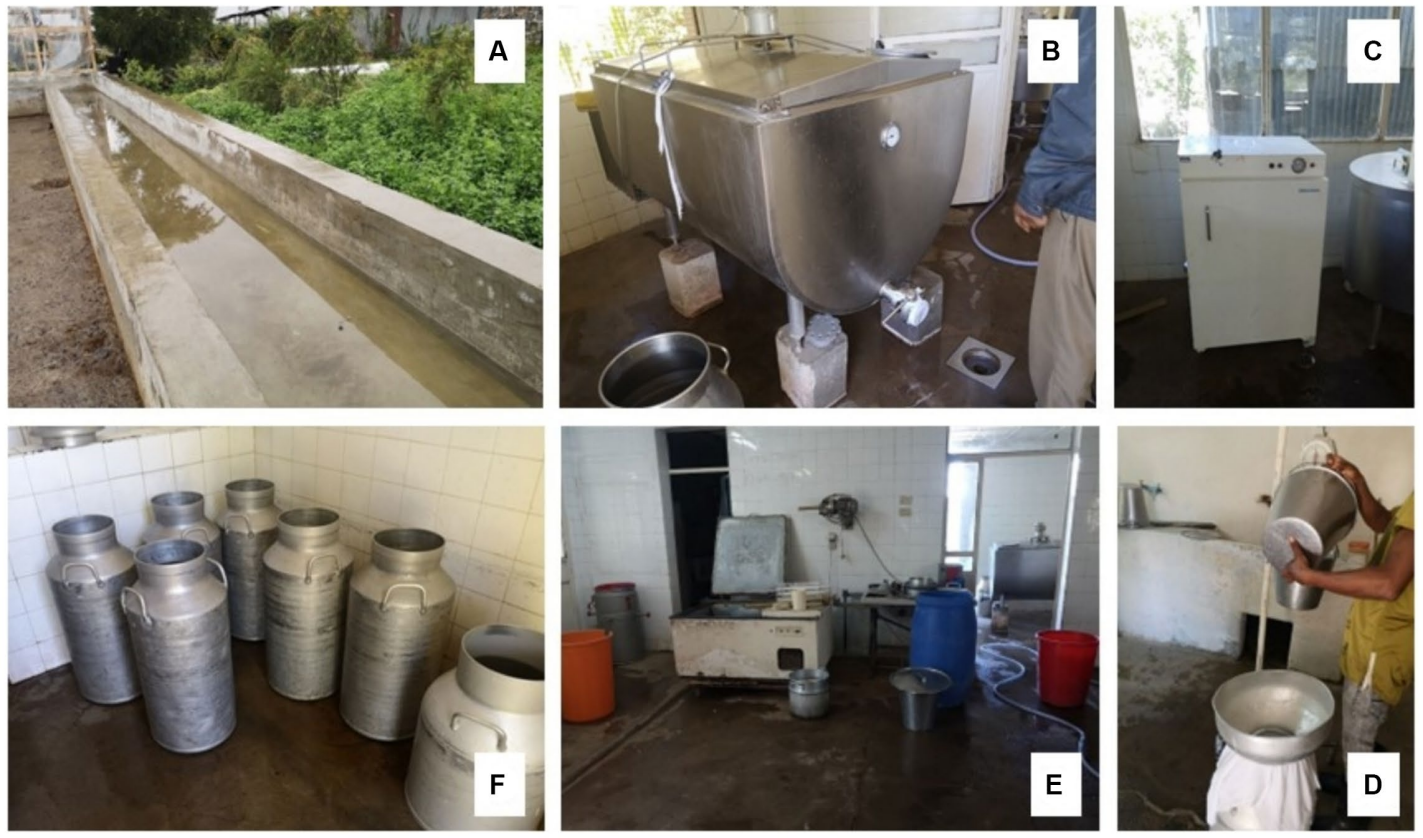
Improved cattle water trough and improved milking and storage equipment. **(A)** Improved water trough; **(B)** Milk cooling tank; **(C)** A fridge for storing drugs and samples; **(D)** Milk weighing and sieving to ensure quality; **(E)** Milk processing unit in a big farm; **(F)** Aluminum milk storage containers.

Table 1 summarizes the characteristics of the PD dairy farms included in this study and the technologies adopted and modified by PD farmers to ensure that these innovations were suited to their specific context. The practices and technologies adopted in farms visited for participant observations are summarized in Table 2. Examples of these technologies and innovations are presented in Figures 1–3. Technology adoption was driven by PD farmers’ entrepreneurship orientation, proactiveness, innovativeness and risk-taking behavior.

**Table 1:** Summary characteristics of participating farms.

Locations [*n* (%)]	Addis Ababa	16 (48.5%)
	Oromia	17 (51.5%)
Cattle breed [*n* (%)]	Crosses with exotic breeds	20 (61%)
	Exotic breeds	12 (36%)
	Both local and exotic breeds	1 (3%)
Number of cows in the herd (herd size) (mean Std ± dev)		Mean 33 (±22) Range 11–99
Number of lactating cows in the herd (mean Std ± dev)		Mean 16 (±10) Range 5–47
Number of calves in the herd (mean Std ± dev)		Mean 7 (±6) Range 1–26
Milk produced per day in the farm (in liters) (mean Std ± dev)		Mean 120 (±86) Range 15–342
Where milk is sold [*n* (%)]	Traders	22 (66.7%)
	Dairy processing companies	2 (6.06%)
	Coops	8 (24.24%)
	Consumers	20 (60.6%)
Breeding method [*n* (%)]	AI	20 (60.6%)
	Bull	3 (9.09%)
	Both AI and Bull	10 (30.3%)
The farm has a disinfection bath (biosecurity measure) [*n* (%)]	Always	5 (15.2%)
	Sometimes	4 (12.1%)
	Never	24 (72.7%)
Purchase modern commercial feeds and minerals [*n* (%)]		33 (100.0%)
Conserve crop residues from your farm or buy and conserve them [*n* (%)]		20 (60.6%)
Vaccinate your cattle to prevent diseases [*n* (%)]		31 (93.9%)
	Biogas	4 (12)
What do you do with farm-generated manure [*n* (%)]	Spread manure on grass/pasture grown for cattle feed	5 (15.2%)
	Spread manure on grass/pasture not used as cattle feed	14 (42.4%)
	Cow dung patties/briquettes	6 (18)

**Table 2:** Participant observation of positive deviant behaviors and technologies (*n* = 15).

Farm	Feed mixing	Growing own improved feeds	Feed conservation	Cattle vaccination	Use of AI	TB testing	Good manure management	Improved housing	Use of new equipment/machinery	Milk value addition	The farm has quarantine and isolation units	The farm has a footbath
1	x	x	x	x	x		x	x	x			
2	x	x	x	x								x
3		x	x	x								x
4	x	x	x	x	x		x		x		x	
5				x								
6	x		x	x	x							
7	x			x			x					x
8				x			x					
9	x	x		x								x
10	x	x		x								
11	x		x	x				x		x		
12	x			x	x							
13	x			x								
14				x								
15	x		x	x								

#### Farmers breed selection and use of artificial insemination (AI)

PD farmers, driven by their entrepreneurial and risk-taking behavior, adopted innovative technologies to increase milk production, including the adoption of artificial insemination (AI), sometimes with sexed semen. However, none of the farmers had yet adopted the more expensive and more technical embryo transfer technology. The majority of the PD farmers in urban and peri-urban areas had adopted highly productive exotic breeds, mainly crosses of Holstein Friesian, Ayrshire, despite recognizing that these breeds required more care than local breeds, including improved housing, feeding, animal health care and hygiene, to ensure they were productive. Two of the PD farmers in peri-urban areas kept local cattle breeds which produced lower levels of milk but were adapted to local environmental conditions, such as grazing on low-quality grasses, and were resistant to endemic cattle diseases.

“[We tested our cattle 5 years ago] *they were 65%-75%* [Holstein-Friesian] […] *and they said the breed will keep getting better over time to time* […] *you can see the changes even in their physical appearances and performances, that they are getting closer to 100%*” Farmer 1.

*“We added one exotic breed with 95% exotic level* [bought from a breeding company; Holland dairy] *The cow was giving 30 litres per day* […] *we have added new cows and bred them, we now have their descendant which are giving 20-22 litre per day* […] *However two years ago we moved the farm* [and] *milk production decreased because the cows do not get enough feed*” Farmer 2.

PD farmers had adopted artificial insemination (AI) as their main breeding method to maintain breed quality. The cost of AI ranged from 100 birr^2^ for government-provided services to 150 birr for private animal health service providers.

*“I was using a bull, and there were not enough professionals for artificial insemination as they are now. But when breed quality and productivity got lower over time* […] *I was forced to shift to AI from a supplier company called Alps* [private company]*. Nowadays, there are better breeds from the government as well”* Farmer 1.

There were challenges associated with AI services, with the majority of PD farmers reporting that they had experienced AI failure, which had led them to resort to using their bulls for mating.

“*We have twenty calves, from those we got only two of them with AI, others we got from bull breeding* […] *We got from the farm by selecting our best animal* [bull]” Farmer 2.

Private animal health service providers are helping PD farmers in their efforts to adopt new technologies, such as the use of AI, by providing services and advice. These business relationships could, however, result in technology and business lock-ins (i.e., farmers being dependent on service providers and their services) which could stifle on-farm experimentation and the ability to try new ideas or technologies.

“*They asked us to inform them if we have problems with AI services* [failed AI], *and a repeat will be offered free of charge. It was only 15 birrs* [paid for one insemination]. *Then it was raised to 25 birrs* […] *there are also other expenses to get a professional* [mainly transport]” Farmer 3.

Although the cost of private services was perceived as high, the quality of their services was deemed to be better compared to government-subsidized services. Private AI service providers arrived on time and were perceived to have fewer AI failures compared to their government-funded counterparts.

“*Private sector insemination services cost more and are better compared to public services* [government-provided and often subsidised service]” Farmer 4

#### Farmers’ adoption of vaccination and biosecurity measures

Cattle diseases were reported by PD farmers as being common in the crowded zero-grazing units associated with small dairy farms. There was low adoption of biosecurity measures, which exposed cattle to disease risks (see Table 1). PD farmers explained that it was easy for disease transmission to occur between cattle within the zero-grazing herds due to close contact in the limited space.

“*Our cattle suffer from foot and mouth disease (FMD) and pneumonia, which doesn’t get enough attention from the agriculture office since it occurs less frequently* […] *Mastitis occurs more frequently*”. Farmer 6.

All of the farmers interviewed had experienced mastitis within their herds, a disease that had a significant impact on their income and livelihoods as sick cows reduced milk production, and their milk had to be discarded. Mastitis cases were regarded as being a consequence of poor housing and hygiene conditions, characterized by wet floors in the shed where cattle slept or rested.

“*Two cows have been affected by it* [mastitis]. *We have taken the appropriate measures for the sanitation problem.* [It is difficult] *to get medications for mastitis in the market. There was a medication called Lacta which is administered through their teats, but we can’t find it now in the market, we are using Gentamycin and Penstrip instead*” Farmer 1.

Farmers actively addressed cattle disease risks through regular vaccination to prevent disease risks and also cleaned the cattle sheds to keep pathogens off and prevent the introduction of diseases into their farms.

“*We regularly vaccinate for 5 different diseases, including FMD and Black Leg”* Farmer 9.

“*Their* [cowshed] *is cleaned with a disinfectant every 2 weeks*” Farmer 1.

Moreover, nearly half of the farmers reported that they had asked their veterinarians to change their clothes and use shoe coverings before accessing their cow sheds.

“*When* professionals [vets and extension officers] *come, they change their clothes and cover their shoes [often with plastic bags] we have a disinfectant solution to step in for shoes*” Farmer 13.

“*There are airborne diseases as well as those transmitted through the shoes of individuals who come in contact with the cows. I think professionals primarily must take care. They have to teach while they are giving services in different farms, since farmers may not be aware*” Farmer 1.

#### Farmers’ adoption of feed production, conservation, and feeding practices

All of the PD farmers reported that cattle feed was the major challenge they faced. Seasonal feed availability compromises dairy production in smallholder farms. The majority of the PD farmers had small farm sizes that could not support the production of cattle feed. Eight of the 15 PD farms visited for participant observation had larger farm sizes in the peri-urban and rural areas and grew feeds such as Napier grass, alfalfa, grasses (boma Rhodes–*Chloris Gayana-cultivar* ‘*Boma*’ and *Bracharia* spp.), and silage maize. Two of the farmers had boreholes that allowed them to irrigate their forages, including Napier grass, alfalfa, and silage maize (see Figure 1). Large farms that grew their fodder and forages did not face the seasonal feed shortages experienced by small farms.

Farmers with small parcels of land bought and conserved feeds, including hay and different crop residues such as teff, barley and wheat straws when they were abundant and cheap. Farmers conserved feeds in modern stores or through traditional means, such as piles kept in the field or farm compound, often exposed to weather elements (Figure 1). The majority of PD farmers purchased brewers spent grain, flour mill by-products such as maize bran, wheat bran, oilseed cakes (Noug seed cake, linseed, sesame, and rapeseed), sugarcane by-products like molasses and bagasse and grass hay, Napier grass and native grasses that are then chopped and mixed at home to create a balanced tailored ration for high producing cows (Figure 1). However, farmers perceived the price of feeds to be expensive and significantly impacted their profits.

“*We are suffering from expensive animal feeds* […] *dairy business is not profitable nowadays* […] *since we provide our animals with good feeds, they do not get sick easily*” Farmer 1.

“[feeds] *are too expensive. For example, the price of Furshka (a ground mix of cereals) was around 500 birrs for 100 kilos. Now it is up to 1,700 birrs. In the past, we were able to buy 100 kilos of beer sludge* [brewers spent grain] *for 100-120 birrs; now it is being sold at 250 birrs. So, it is getting harder and harder*” Farmer 13.

Farmers felt that farm-mixed rations cost less and provided their cattle with better nutrition compared to commercial concentrates. Moreover, they were aware that poor feed quality affected cattle health and that “bad feeds led to cattle illness while good feeds led to good health and improved production.” Farmers had learned through trial and error and figured out the ratios that were appropriate for the cattle’s nutritional needs. One of the PD farmers had a container that she used to measure how much feed each cow was to receive, depending on the lactation stage. This innovative approach was appreciated by other farmers in the area who visited this farmer for training on feed preparation, mixing and feeding strategies for their cows.

“*We give feed based on their production stage, and we record it. For example, if a cow gives 12 litters per day, we give it 6 kilos of feed*.” Farmer 15.

#### Farmers’ adoption of improved cattle housing

The design of cowsheds and the space available for cow movement were highly influenced by land size, especially in urban and peri-urban areas. Small land sizes in urban areas constrained PD farmers’ ability to expand and improve their cowsheds due to a lack of space to build. Only the big farms in rural areas with space allowed their animals outside to graze and/or rest (Figure 2). The majority of smallholder farmers in urban areas practiced zero-grazing, with cows being kept in barns either tethered or untethered. Poor housing conditions in these zero-grazing cowsheds contributed to poor animal welfare due to the low space available for cows to move around. Zero-grazing cowsheds contributed to diseases such as mastitis and bovine tuberculosis due to the unhygienic housing environment and lack of ventilation. The majority of the cowsheds were improvised and constructed using mud or iron sheets for walls and had earthen floors.

“*This is where we live, and this is their house* [sharing a home compound with cattle]. *What we do is prohibit kids from coming out* [into the compound to play] *and bring out the calves and pregnant cows to get fresh air and to have a walk to stretch their legs. In comparison, mine* [farm] *is even bigger than other farms in the area.*” Farmer 1.

Tethering cows to one spot led to ill health and health complications among cows, including arthritis, body wounds, sores and mastitis. PD farmers used wood shavings and sawdust to increase cow comfort and to dry the wet floors.

“*It was challenging in the past when they were sleeping on plain cement floors. But since I started to put down chipped wood* [locally called Segatura] *I get from furniture makers on the floor and cleaning the floor regularly, the problem is now gone. They were getting the disease [lameness and body sores] because of the cold, wet floor and poor hygiene of the floor*” Farmer 9.

However, large farms with access to financial resources had constructed modern cowsheds that ensured cows had good animal welfare due to the spacious conditions and easy-to-clean concrete floors (Figure 2).

#### Farmers’ manure management practices

Manure management was a major challenge for farms in urban and peri-urban areas due to land scarcity resulting from land subdivisions for housing development and rapid urbanization. Manure disposal was perceived to be an environmental problem due to risks of pollution, especially for rivers and streams and the environment, which could spread cattle diseases. Smallholder dairy farmers with small land sizes struggled with manure disposal. Farmers dumped manure by the roadside, in open fields or near rivers and streams. Some farmers, however, had developed novel ways of utilizing cow dung as manure to grow cattle feeds and food crops. Ten farmers reported that they made cow dung “*briquettes and patties,*” which were then used as fuel for cooking. Two farmers burned cow dung in the cattle shed as an insect repellent. One farmer had constructed a concrete pit where cow dung was accumulated and then disposed of by giving it to tree nursery operators once the pit was full, once every 3 or 4 months. One farm had a biogas plant whereby manure was used to produce biogas used as cooking fuel (Figure 2).

“*We don’t have space. In the past, there were plenty of fields in the area. Now, the local administration prohibits us from using the plain fields. It is not only me but others in the area as well who are having difficulty even disposing of their cow dung*” Farmer 18.

#### Farmers record-keeping practices

All PD farmers kept records mainly in the form of notebooks, although some farmers are adopting new means of record-keeping, such as online record-keeping apps. Farmers kept records of milk production, feeds, AI dates, calf dates of births, and vaccinations, among other farm costs and activities.

“*It is hard to be productive without keeping records in this business* […] *I record the date of insemination, when a calf is born, and the age of the heifers*” Farmer 1.

Five farmers performed ear tagging of their newborn calves and registered them with the breeding organization, which added value to the potential sale of these animals. Ear-tagged and registered cattle were recognized as attracting higher prices in the market.

“*Yes* [all our cows are registered] *have* [ear tags] *but some calves born recently did not have them yet, although we have applied* [ear tags for registration purposes] *for them*” Farmer 5.

#### Farmers’ groups and cooperatives membership

PD farmers formed farmers’ groups and cooperatives to harness the benefits of collective action. Through these farmer groups, farmers organized to have all the cows belonging to their members vaccinated on the same days, which ensured that vaccination was done cheaply, and no vaccines were wasted. Procurement of services, such as the supply of vaccines, through farmer groups and cooperatives allowed farmers to establish business arrangements that enabled them to enjoy economies of scale and ensured reduced transaction costs. Animal health providers were able to serve many farmers and animals in the same area, which reduced the transaction costs associated with animal health services (i.e., transport costs).

“*We have a dairy owners' association in our sub-city. It was established due to the scarcity of vaccination professionals* […] *we formed it to tackle that problem together*” Farmer 1.

“*We were facing these challenges* [in farm production]; *we were forced to form an association* [that has allowed us] *and give vaccinations. We are functioning without waiting for the government’s assistance but still working with government animal health professionals in the sub-city. If we wait for the government’s assistance, our cows will be in trouble* […] *We coordinate vaccination programs for those cows that didn’t get the vaccine the first time it was given*” Farmer 2.

“*We vaccinated our cattle annually twice before we came to here* [Bishoftu]*. We could not get that service after we moved here. When we asked them, they responded that they were not going to open vials for a few animals* […] *there was a problem with animal vaccination*.” Farmer 4.

Farmer groups also allowed PD farmers to aggregate their milk and sell it in larger quantities to processors, which reduced the transaction costs associated with bulking small quantities of milk with heterogeneous quality influenced by farmers’ hygiene and handling practices. Farmer groups also ensured that all the collected and bulked milk met the expected quality standards, which ensured access to the formal milk marketing channel that involved large processing companies.

### Drivers of positive deviance in smallholder dairy systems

Several intrinsic and extrinsic factors influenced PD farmers’ behavior as regards adopting, modifying, and using technologies and innovations. These factors include personal attributes (e.g., risk-taking propensity and entrepreneurial behavior), socio-economic characteristics, cultural norms, and market and institutional factors.

#### Intrinsic drivers of positive deviance in dairy farming systems

Intrinsic factors are an individual’s attributes that influence their decision-making behavior and actions. These factors include educational level, financial and resource endowment, gender, and risk propensity.

#### Learning from experience

All of the PD farmers relied on their lived experiences to inform their future farming decisions. Farmers had recent proximal experience with livestock diseases and recent memories of disease outbreaks. They were able to identify the diseases their cattle had contracted in the recent past, and they knew about recent and past cattle disease outbreaks including incidences of foot and mouth disease (FMD), milk fever, brucellosis, bovine tuberculosis, mastitis, hoof diseases, arthritis, dermatophytosis, blackleg, lumpy skin disease (LSD), pasteurellosis, tick infestations, anthrax, contagious bovine pleuropneumonia (CBPP).

“*Previously, blackleg, pasteurellosis, and LSD were the problems, and the burden of blackleg and pasteurellosis was lowered by the vaccination program. I know LSD is still a problem even with ongoing vaccination efforts*” Farmer 22.

As a consequence of their lived experience, PD farmers took action to minimize cattle disease risks and increase resilience of their farms to external shocks, such as vaccinating cattle, improving feed and housing, and improving hygienic practices.

“[Learning] *is through my efforts. I read from publications* [such as] *magazines or flyers produced by the Ministry of Agriculture. The other source of information is from animal health professionals and animal production experts at the sub-city or district level* […] *reading and consultation with professionals are my main sources of information*.” Farmer 29.

#### PD farmers’ risk-taking behavior and long-term strategic business thinking of incremental growth

All the PD farmers had started as smallholder dairy farmers with just a few cows and grew their farms and herds over time. PD farmers regarded themselves as entrepreneurs who had to bear substantial business risks in the present and expected to earn a return on investment in the future. They described themselves as curious people with problem-solving capacities that allowed them to face challenges faced during their day-to-day dairy farming activities. They had financial resources that cushioned them from losses and were more willing to take risks compared to their peers who did not have access to capital or insurance mechanisms, including access to financial resources from financial institutions, immediate family members and people within their social networks. They were willing to invest in high-producing cows, vaccines, housing, and improved feeds to increase their milk production and reduce cattle disease pressure. By being innovative and risk-taking, they were able to increase their profit margins and also reduce the probability of incurring losses. Farmers were willing to experiment with and adapt innovations and technologies to fit their context-specific needs and the realities of their farms.

“*It has been around 17 or 18 years since I started. I chose this sector after seeing its profitability from other people, even though it is a challenging one.*” Farmer 29.

PD farmers believed that, although they faced challenges in the short term, they had the skillset and patience to become successful entrepreneurs in the long term. Their strategic thinking mindset allowed them to make investments in technologies and innovation with the expectation that they would recoup their costs and get returns in the future. They invested prudently in the growth of their farms, starting with a few animals and growing their herd and production capacity over time.

“*Before you even think of capital, you have to consider some preconditions* [prerequisites]. *You have to consider whether the area is suitable for cattle production or not, and whether there are ways for cattle waste disposal. Then you have to consider how many cows you have to start with, taking the space you have into consideration*” Farmer 29.

“*Initially, I had no capital. I started with one cow that I bought for 4500 birrs in 2012, which gave me 12 litres per day. The next year, I added another pregnant heifer that cost 8000 birr. At that time, the community feared exotic breeds and complained about their feed consumption. Now I have a car, a good living house, and several more cows*” Farmer 33

#### Extrinsic drivers of positive deviance in dairy farming systems

There are several external factors that shaped farmers’ PD behavior, including social norms, culture, customs, social networks, institutional factors such as extension, animal health, access to credit and market.

#### Social and cultural norms

Social norms play a key role in agricultural entrepreneurship in Ethiopia. Dairy farming has a symbolic and cultural meaning not only as the primary source of food and livelihood for farming households but also in shaping social-cultural values embedded in the local social norms. PD farmers reported that dairy farming directly and indirectly provided employment opportunities to people working in the dairy value chain and contributed to economic success and prosperity in rural areas. Shared grazing areas, water resources and local knowledge, particularly in rural areas, enabled the creation of a thriving practice of commensality (i.e., sharing of food and eating together) that led to stronger communal social bonds and ties within the local communities.

“*I do not have training in this business* [dairy farming]. *I started it out of my desire to continue a way of life. I became experienced over time* [in dairy farming]” Farmer 1.

All of the PD farmers reported that dairy cattle constituted a popular asset in rural areas and conferred on farmers a social status, identity and prestige associated with possessing a herd of cattle. Moreover, dairy cattle play important societal roles, such as payment of dowry and a store of wealth that families can draw upon in times of need. Although uncommon in the current mainstream financial system, dairy cattle or milk sales are accepted as collateral for loans, particularly in farmer groups and savings and credit cooperatives (SACCOs).

Dairy farming is an important form of transmitting traditional knowledge and values, such as working hard, unity and equity, from generation to generation and as a way of keeping the culture and traditions alive in Ethiopia’s broader society. Dairy farming enables women to partake in economic and entrepreneurial activities, which enable them to earn income through milk sales and value addition activities, which contribute to the reduction of gender inequalities within the broader society. Furthermore, dairy production contributes to communities’ food security and also sustains local traditional food cultures and knowledge through the production of local food products of cultural value, such as artisanal cheeses and yoghurts, that are sold and/or shared with family and friends.

“*I do it all alone* [farm work] *for all of the 14 cows. A physically weaker person will be challenged to do it since it requires muscle power. We keep doing it since we don’t have any other alternative* [since it is my main source of livelihood]” Farmer 16.

#### Access to information, social networks, and peer-to-peer learning

PD farmers were willing to invest money in acquiring information from veterinarians, professionals in the industry and their peers. Access to information was crucial to shaping farmers’ behavior and influencing their adoption of modern dairy farming technologies. Farmers sought to learn about modern technologies and practices from fellow farmers. They copied and adapted farming technologies and practices to fit the local context within farms and specific locations.

“*We get information from professionals as well as from other individuals* [farmers] *who are in the business and who are experienced. When we hear that there is a better practice on someone’s farm, we will contact them to ask for their experiences and see if we can learn or copy what they are doing*.” Farmer 16.

Moreover, PD farmers had close relationships with veterinarians and were willing to pay for the services of experts to improve their farms’ productivity. Farmers attended training provided by government agencies, development agencies and Non-Governmental Organizations (NGOs) when such opportunities became available.

“*We learned a lot from our family and experience. I took an animal production training course at the beginning of my school studies. I also use the internet for information by googling*” Farmer 5.

Searching for animal health and advisory experts attracted extra costs. However, PD farmers were willing to incur these costs to increase productivity and prevent losses, particularly from cattle diseases.

“*When we encounter problems, we call vets, and they will tell us what to do*” Farmer 13.

Farmer groups and cooperatives provided PD farmers with the opportunity to access information, technologies such as aluminum milking and storage containers and training that enabled them to make informed decisions. Access to information was perceived as minimizing the risks associated with technology adoption as it allowed farmers to assess the costs and benefits of dairy technologies. Social networks within farmers’ cooperatives and groups facilitated peer-to-peer and social learning and allowed farmers to learn from each other. This allowed farmers, especially more risk-averse farmers, to copy practices and technologies that were already successful.

“*One benefit* […] *is that we are a partner with Alema Farms* [a joint venture between a local investor and investors from the Netherlands]. *They give priority to those buyers who come as a cooperative rather than individual buyers* […] *we get a 30 birrs discount for every 100 kilos of feed we purchase. That discount helps us to cover transportation costs*” Farmer 3.

However, cooperatives and farmer groups struggled due to differences of opinions, member interests and governance challenges in cooperative management, which led to their failure and collapse. Low membership numbers in farmers’ cooperatives denied farmers the opportunity to enjoy economies of scale related to bulk purchase of feeds and animal health services.

“*Age has its impact, I think. Many of our members are old, and you see reluctance and rigidity to adopt modern technologies or beneficial ideas*” Farmer 1.

“*Many think working alone is better, rather than cooperating and collaborating. Even though there were 53 members when it was formed, currently only 14 members are left*” Farmer 3.

#### Institutional issues

Farmers relied on animal health and extension professionals for information and assurance that they were making sound decisions, with animal health professionals seen as informed social referents. Low supply of medicines and vaccines, attributed to low government funding for public animal health clinics, however, created tensions between farmers and animal health professionals.

“*Vaccines are manufactured in Debre Zeit, and many times it is hard to get them* [due to shortage]*. Their manufacturing time* [is not aligned] *with seasonal disease outbreaks. Cattle diseases are seasonal* […] *like when fresh grass is purchased and brought to the farm* [or], *when seasons change, and stagnant water occurs* […] *diseases can be brought through people’s shoes*.” Farmer 5.

PD farmers felt that the government and its institutions did not take smallholder dairy farming seriously and did not prioritize solving farmers’ pressing challenges, despite the sector contributing to employment and food security.

“*In addition to being a challenging sector,* [livestock farming] *does not get [the] government’s attention*” Farmer 1.

Farmers alluded to the fact that important animal health clinical supplies were often not provided by the national and local governments, which hindered the provision of adequate animal health services at the local level.

“*Sometimes they run out of liquid nitrogen and semen, and as a result, we miss the chance of conception of our animal*” Farmer 5.

“*When professionals come from government clinics, they will bring medications and give services. But if the* [livestock disease] *case needs a prolonged follow-up* [we are forced to buy the supplies and medicine] *since such provisions are not provided by the government*.” Farmer 1.

Moreover, farmers were constrained by government bureaucracy, which made accessing essential services slow and prohibitive. They reported that decisions regarding access to services were made behind closed doors and that it often took a long time to get feedback from service providers, which conflicted with their need to make quick business decisions.

“*The responsible bodies of the government, for example, district-level executive bodies, don’t allow for the farmers to expand their farms [*…*] There is a lengthy bureaucracy and procedures. It is hard to keep the sanitation of cows on the farm in such instances*” Farmer 21.

#### Market factors and milk value addition

Market factors influenced PD farmers’ entrepreneurial behavior and decisions. Given the investment needed to start a farm and invest in improved technologies and innovations, PD farmers believed low milk prices were a disincentive for those looking to enter into farming and/or expand their farming operations. Milk processing was highly seasonal, and prices were low in the rainy season and the fasting period of Orthodox Christians. To cope with seasonal variations, entrepreneurial PD farmers, through farmer groups, processed milk into artisanal cheese, yoghurt, and butter, which they sold locally. Value addition activities such as milk processing were particularly important during the fasting season when the majority of Ethiopians, who are members of the Ethiopian Orthodox Church, abstain from the consumption of animal products.

Although compliance with good agricultural practices was better in PD farms, enforcement of milk quality standards at the farm level was low. In the absence of a quality-based payment system, PD farmers who invested in improving their milk quality were not rewarded for their efforts and investment in improved milking and storage equipment and technologies, e.g., aluminum containers, milking equipment, milk cooling tanks, etc. (Figure 3). Farmers found it hard to access credit from financial institutions, especially banks, using their livestock or milk sales as collateral. Only five farmers practiced improved milking and handling hygiene practices, such as sieving milk to remove hair and other physical debris before selling it unpasteurised to consumers (Figure 3). The majority of milk produced by PD farmers was directly sold to consumers. Two farmer groups processed milk into artisanal cheese, yoghurt, and butter, which they sold locally, creating employment opportunities for local communities, especially women and youth. Larger farms sold their milk to informal traders or processors. Milk quality testing was not, however, common, and the quality of milk received by buyers was based on trust between the farmer and buyer.

“*It is based on trust* [milk quality]. *People traditionally identify the quality of milk through its thickness and thinness. If it is thicker, it means it’s oily* [fatty] *in nature. Consumers benefit and enjoy it* [nutritionally and taste]. *You can manage to maintain milk quality by providing appropriate feed to your cows* […] *I have learnt all that from my own farming experience*” Farmer 1.

#### Environmental factors influencing dairy farming

Environmental factors such as climate change, including increasing episodes of drought and low rainfall, have exacerbated feed shortages and caused reduced milk production in Ethiopia. Poor harvests due to a lack of rainfall have driven up the prices of feeds, which have increased production costs and reduced farm revenues. Declining farm sizes render manure disposal challenging, which makes it difficult for new entrants into dairy farming. Urban development has adversely impacted the amount of land available for grazing and access to farmland and farm sizes. Urban residents’ complaints about the smell of manure and noise emanating from dairy farmers have also led to new restrictions being placed on farms. These factors could constrain farmers’ adoption of technologies and innovations, their ability and willingness to invest in dairy farming, and increase the risk of losses for farmers, forcing them to exit the farming sector.

## Discussion

This study explored PD farmers’ entrepreneurship behavior and their adoption and use of agricultural technologies and innovations in Addis Ababa city and the Oromia region in Ethiopia, through a qualitative research approach. We observed that PD dairy farmers were driven by profit motives, social status, risk-taking behavior and ambition to grow. The results of this study reveal that entrepreneurial PD farmers have devised novel strategies that have enabled them to have better livelihood outcomes through improved farm productivity, animal health and welfare (5, 12) despite the sub-optimal performance of dairy production systems in Ethiopia (2–4). This study shows the imperative for policymakers and dairy-sector stakeholders to acknowledge, in the design of relevant policies and intervention strategies, farmers’ agency and their existing capacity to find solutions to the challenges faced. PD farmers are proactively addressing challenges such as feed shortages and livestock diseases through feed conservation, growing fodder and procuring animal health. Policymakers and dairy-sector stakeholders should, therefore, look to address the systemic barriers that farmers face and build on their existing capacities; empowering farmers through the provision of context-specific training and ensuring that interventions, technologies and policy changes are inclusive, viable and practical for the local contextual realities (22).

The results of this study show that PD farmers have managed to find solutions to day-to-day dairy production challenges faced through their adoption of technologies and innovations. Their creative use of knowledge has played an important role in the innovation processes and provides solutions adapted to local contextual realities (13). PD farmers’ practices derived within the local biophysical landscape using available resources have long-term sustainability potential (3). These technologies and practices have the potential for easy diffusion and sustainable long-term uptake within the dairy farm community (23).

### The promise of positive deviance in solving dairy farming challenges in Ethiopia

This study reveals that successful solutions to address livestock production challenges exist within the dairy community. These findings show that it is possible to address the systemic challenges facing the dairy sector, including low adoption of dairy technologies, low milk production per cow, poor animal health and welfare (3, 4). The results further show that better farm performance can be achieved through innovative use of available assets, inputs and processes, as has also been reported by de Adelhart Toorop (10). Previous studies have reported that the adoption of dairy technologies such as improved dairy cows, adoption of AI services, improved forages and use of veterinary services, is positively correlated with increased farm production and profitability (3). There is, therefore, an imperative to support farmers’ technology adoption efforts as a way to improve farming livelihoods and support rural development.

The results of this study show that PD farmers are addressing animal health challenges through their proactive procurement of animal health services. Livestock production in Ethiopia is constrained by the underfunding of veterinary services, which leads to poor-quality and low coverage of veterinary services (24, 25). Dairy production in Ethiopia is also undermined by the high prevalence of endemic cattle diseases such as mastitis, bovine tuberculosis, and brucellosis that thrive in intensive production systems (2, 25, 26). Many of these endemic diseases are zoonotic, exposing farmers, farmworkers and consumers to public health risks (26, 27). Promoting the PD farmers’ animal health-seeking behavior and strategies among the wider farming community can lead to improved animal health and welfare and better public health outcomes (4, 14, 20, 28). The adoption of biosecurity measures can also contribute to improved animal health and welfare, which can be beneficial for dairy farmers in Ethiopia (7, 29, 30).

The results of this study show that PD farmers adopted and modified technologies to address the contextual challenges they face, including feed access challenges, small land sizes and low access to capital to invest in expensive machinery. This research is in agreement with previous research that reports that feed availability and quality are a major constraint for dairy production, especially in urban and peri-urban areas, where there is limited land availability, and farms are small in size to grow fodder (8, 31). The findings of this study on how dairy farmers have innovated on dairy ration preparations using locally available resources such as teff stover and other industrial by-products are in agreement with the findings of (32). Moreover, PD farmers have experimented through the trial-and-error approach and figured out the sufficient ration volume to be fed per animal to ensure they keep their production costs low and maximize their income. However, poor feed storage and conservation could expose feeds to contamination, particularly aflatoxin contamination, which is detrimental to animal health and welfare, food safety and dairy production (8, 20).

### Drivers and constraints for positive deviance in smallholder dairy systems

The result of this study shows that PD farmers, through their innovation capacity, have the agency to successfully tackle dairy production challenges. Farmers learn through doing, researching, interacting and solving their challenges, which goes to show that an individual’s learning capacity is crucial for innovativeness and entrepreneurship (4, 13). Learning is the capacity of an individual to act based on the resources and competencies, such as skills, material, and financial resources, available to them (13). PD farmers can serve as social referents, role models and as a crucial source of information that could aid their peers in decision-making and facilitate technology diffusion within the wider farming community (3, 13).

The result of this study shows that farmers adopt technologies or modify them to suit their contextual farm factors, driven by intrinsic or extrinsic factors. It is, therefore, crucial to note that farmers’ awareness of a technology or innovation does not automatically translate into wide-scale adoption; rather, this is contingent on a technology or innovation fitting within a farm’s given context (13). PD farmers’ innovation is often initiated spontaneously at the farm level based on observations and a trial-and-error approach to experimentation (13, 33). PD farmers are pioneers on the technology adoption curve, and their success can underpin more widespread technology adoption and diffusion across farming communities (33). PD farmers use their capacity, resources, assets and problem-solving skills to challenge existing organizational structures and the status quo (33). PD farmers employ innovative practices and strategies which lead to successful outcomes despite having similar resources and facing the same barriers as their peers (3, 13). PD farmers’ technology adoption is influenced by their knowledge, attitudes, and biophysical and socio-economic environment (13). Previous research shows that the availability of off-farm income enables PD farmers to invest in technologies that enable them to take the risk associated with the loss of income if the adopted practices or technology fails (3, 13, 23).

### Policy implications

Positive deviance research method can contribute to addressing complex societal problems by uncovering solutions that already exist in society (23). Positive deviance research method can be used to select a subset of efficient practices for scaling up from the multitude of practices adopted by farmers (10). PD farmers can support their peer farmers in decision-making and can also act as trainers of the dairy farming community (33). Rather than look for ‘socio-technical fixes’ and innovation solutions from outside a given farming community, researchers must facilitate farmer-led or farmer-centric innovation and recognize that viable solutions may already be found within the local communities (10).

The findings of this study show that farmers are willing to adopt improved breeds and the use of AI, which can improve animal production (3). However, there is a need to also provide farmers with knowledge and support the adoption of related technology that is needed to realize the milk production potential of improved dairy breeds, such as improved forages, record keeping, veterinary services, improved housing, biosecurity measures and animal welfare (3). Moreover, the government and related stakeholders can disseminate and promote successful technologies and practices used by PD farmers, such as the adoption of biosecurity measures to control disease risks, which could improve dairy farms’ productivity (34, 35). Finally, there is a need to support farmers in urban and peri-urban areas with effective farm-waste management by promoting waste valorization through circular business models and developing new uses for manure (36).

The results of this study highlight the positive role of peer networks and trust among PD farmers, particularly in farmers’ cooperatives and groups. There are opportunities to leverage PD farmers as “champions” and change leaders (10). Moreover, there’s potential to design peer-led demonstration programs and use them as “model farmers” for technology diffusion (10, 36).

Farmers are by nature averse to risks associated with adopting dairy technologies (3). It is important to understand how PD farmers are able to take business risks, how they raise capital and how they safeguard themselves from financial losses. This study shows that PD farmers follow a piecemeal adoption strategy, starting small, learning on-farm and then scaling successful ideas or technologies as a mechanism to cope with and/or minimize risk exposure.

Findings of this study highlight the institutional constraints that farmers face, including low access to credit, animal health services, extension support and farm inputs. By understanding how PD farmers overcame such barriers, policymakers can design policies that support the growth and sustainability of the dairy sector. There is a need for policies that provide entrepreneurial support for farmers that can support farm expansion and scaling and diffusion of successful technologies through the provision of access to finance, linkage to markets and value chain integration.

The results of this study show a gap in the quality of services provided by government institutions and the challenge related to the high cost of accessing inputs and services from the private market, which constrains farmers’ ability to access inputs and services needed to boost farm production. The current low government resources allocated to improve the quality of supporting infrastructure (e.g., road, electricity, milk cooling infrastructure etc.), and low funding for services such as extension, breeding (AI), animal health and disease control, milk cooling infrastructure, and credit, has been reported as undermining the efforts to improve the productivity of smallholder dairy farming and as hampering farmers’ long-term investment in the dairy sector (3, 4).

The results of this study highlight the scope to increase farmers’ trust in government-provided information and also increase funding for public service provision, including extension and animal health services in Ethiopia. This finding is in agreement with de (10, 13). It is imperative that government and private institutions in Ethiopia, such as financial and research institutions, take steps, such as increasing access to financial and insurance services, to better serve farmers and unlock their potential to take risks and innovate. In urban and peri-urban areas where government services are currently lacking, private institutions have a particularly important role to play in bridging the gaps in service provision.

By supporting dairy farming to a greater extent, government and private institutions could contribute to dairy farmers’ improved livelihood security, while creating employment opportunities across Ethiopia’s dairy sector, and to improved nutrition and public health at a societal level. Institutional changes could support PD farmers’ access to crucial inputs and services that can facilitate their experimentation with technologies and innovations in addressing the challenges faced in pursuing dairy production; this experimentation could contribute to enhancing the resilience of the broader farming community as PD farmers share experiential knowledge and recommend practices to their peers (10, 13).

The findings of this study yield policy lessons that apply to policymakers and dairy-sector stakeholders in countries beyond Ethiopia. There are opportunities for other countries to use the positive deviance approach to identify PD farmers who can act as demonstration farms or mentor farmers for their peers. Moreover, there is a need to support social learning strategies such as farmer field schools and peer exchange as fora to share lived experiences and facilitate the spread and diffusion of good practices and successful technologies. Policymakers can use existing social networks, such as farmer groups, to reinforce trust and knowledge sharing among farmers. There is, however, a need to interrogate the sustainability of the technologies adopted by PD farmers and explore whether farmers maintain high adoption over time, and whether they continue innovating even when they are successful. Finally, there is a need for more research that tracks PD farmers’ technology adoption over time to explore how behavior is sustained and identify place-based contextual factors that can be leveraged to transform the dairy sector (13).

### Limitations

The results of the study should be interpreted in the light of its limitations, which should pave the way for follow-up studies. One of the limitations of this study was a small sample size of PD farmers due to the in-depth qualitative research approach taken by the researchers. Future studies can hopefully focus on exploring bigger sample sizes to understand what happens across the wider farming community in Ethiopia. This study nevertheless provides important insights into the solutions that exist within farming communities, and that can be identified to solve pressing dairy farming challenges. Moreover, the practices farmers adopted by PD dairy farmers as responses to constraints they face could be suitable topics for future research. Building on the findings of the present study, future studies could also explore and compare the farm performance of positive deviants and the control group of farmers. Finally, it is worth noting that practices that may work for PD farmers might not be scalable to less-resourced farmers.

## Conclusion

The objective of this study was to document PD farmers’ existing and emerging technologies and practices and the socioeconomic, personal and contextual drivers of their innovation and use of technologies within farms in urban and peri-urban areas of Addis Ababa and the Oromia region of Ethiopia. The results of this study show that positive deviant behavior results in increased technical efficiency. PD farmers who participated in this study had small but profitable farms despite facing cattle diseases and feed availability challenges. They adopted practices, such as supplementary feeding, cattle vaccination, improved housing, and manure management, learned through observation and a trial and error approach taken to experimentation, from neighbors and relatives, as well as extension and participation in research projects (10). The solutions identified and implemented by PD farmers in this study enabled them to be more successful than their peers and, contingent on their acceptability to the wider farming community, and support received from government and private institutions, have the potential to be scaled up and out and enhance the resilience of the wider Ethiopian dairy sector (13).

## Data Availability

The datasets presented in this article are not readily available because due to the small sample size of this study, the data cannot be shared to protect the privacy of the participants. Requests to access the datasets should be directed to ndungukabi@gmail.com.

## Footnotes

1
https://gtr.ukri.org/projects?ref=BB%2FS013806%2F1

2 Ethiopian birr is equivalent to 0.017 US dollars.
